# The impact of heavy alcohol consumption on cognitive impairment in young old and middle old persons

**DOI:** 10.1186/s12967-022-03353-3

**Published:** 2022-04-05

**Authors:** Fu-Shun Yen, Shiow-Ing Wang, Shih-Yi Lin, Yung-Hsiang Chao, James Cheng-Chung Wei

**Affiliations:** 1 Dr. Yen’s Clinic, No. 15, Shanying Road, Gueishan District, Taoyuan, Taiwan; 2 Center for Health Data Science, Department of Medical Research, Chung Shan Medical University Hospital, Taichung, Taiwan; 3 Center for Geriatrics and Gerontology, Taichung Veterans General Hospital, Taichung, Taiwan; 4 Department of Medicine, School of Medicine, National Yang Ming Chiao Tung University, Taipei, Taiwan; 5 Institute of Medicine, Chung Shan Medical University, Taichung City, Taiwan; 6 Department of Allergy, Immunology & Rheumatology, Chung Shan Medical University Hospital, Taichung City, Taiwan; 7 Graduate Institute of Integrated Medicine, China Medical University, Taichung City, Taiwan

**Keywords:** Dementia, Cognitive impairment, Young old, Middle old, Older adults, Alcohol drinking

## Abstract

**Background:**

Dementia indicates a significant disease burden worldwide with increased population aging. This study aimed to investigate the impact of alcohol consumption on the risk of cognitive impairment in older adults.

**Methods:**

Participants ≥ 60 years were administered the Digit Symbol Substitution Test (DSST) to evaluate cognitive function in National Health and Nutrition Examination Survey (NHANES) cycles from 1999 to 2002 and 2011 to 2014 for enrollment in the present study. Participants were categorized into non-drinker, drinker, and heavy drinker groups. Logistic regression analyses were performed to explore associations between cognitive impairment and alcohol consumption.

**Results:**

Multivariate analysis showed that older adults, men, people from minority races, persons with lower education or income levels, social difficulties, hypertension, or chronic kidney disease were significantly associated with a higher risk of cognitive impairment (all p < 0.05). In the young old (60–69 years), heavy amount of alcohol drinking was significantly associated with lower risk of cognitive impairment compared with drinkers [adjusted odds ratio (aOR): 0.280, 95% Confidence interval (CI) 0.095–0.826]. But in the middle old persons (≥ 70 years), heavy alcohol drinking was associated with higher risk of cognitive impairment (aOR: 2.929, 95% CI 0.624–13.74).

**Conclusions:**

Our study demonstrated that light to heavy drinking was associated with lower risk of cognitive impairment in participants aged between 60 and 69 years, but caution is needed in the middle old people with heavy alcohol drinking.

**Supplementary Information:**

The online version contains supplementary material available at 10.1186/s12967-022-03353-3.

## Background

Dementia is characterized by a progressive deterioration of memory, language, orientation, and judgment that can interfere with daily life [1]. It imposes a significant burden on the patient and his family [1]. Dementia mainly occurs in the elderly, with older adults at a higher risk. Generally, approximately 6% of people aged 65 years and older have dementia, and about 40–70% of people aged 95 years and above have dementia [2]. Due to global population aging, the older population is growing at a rapid rate. The number of people with dementia has doubled from 25.9 million in 1999 to 51.6 million in 2019 [3]. However, there are currently no good options to slow down the progression of dementia or improve its clinical course [1, 4]. Cognitive impairment is characterized by a decline in memory, language, and other cognitive function, which is not severe enough to interfere with daily activities [1]. Cognitive impairment seems to be the prodrome of dementia [4]. Identification of the factors modifying cognitive impairment and interventions may help to reduce the global burden of dementia.

Alcohol is metabolized to acetaldehyde, with direct neurotoxic effects on the brain [5]. Chronic alcohol use can result in thiamine deficiency, leading to Wernicke-Korsakoff syndrome [5]. Animal and image studies demonstrate that alcohol drinking can cause atrophy of the frontal lobe and hippocampus with enlargement of the brain ventricles [6]. However, some observational studies show that light and moderate alcohol drinking may protect against the risks of dementia or cognitive impairment [7, 8]. Some studies show that light and moderate alcohol drinking is associated with a neutral [9] or higher [10] risk of dementia [11] or cognitive impairment [11], but the definitions of the quantity of alcohol consumed, duration, and age of alcohol drinking are different in these studies [11]. Additionally, alcohol use disorders are one of the most prevalent mental disorders worldwide. Yet only about 1 in 6 patients receive treatment. This leaves a large “treatment gap” between the prevalence of the disease and the proportion of patients who received treatment [12].

While most reports about alcohol drinking and cognitive impairment are derived from studies in middle aged adults, to examine the effects of alcohol drinking on cognitive function in older adults seems to be important. As older adults are more prone to underlying brain lesions or lower cognitive function than the general population, which increases susceptibility to cognitive decline after alcohol use [1, 2, 13]. Besides, older adults are more susceptible to the pharmacokinetic effects of alcohol due to decreased lean body mass, a lower percentage of body weight made up of water, and relatively impaired liver function affecting ethanol metabolism [14]. Therefore, we used the data of the Digit Symbol Substitution Test (DSST) [15] from the National Health and Nutrition Examination Survey (NHANES) to investigate the effect of alcohol consumption (at different levels) on the risk of cognitive impairment in persons over 60 years; and also investigated the differences in the impact of alcohol drinking on the risk of cognitive impairment in young-old (60–69 years) and middle-old (≧70 years) people, with consideration of dose effect of alcohol drinking after adjustment of several potential confounders.

## Methods

### Data source

This study used data from the National Health and Nutrition Examination Survey (NHANES) 1999–2002, 2011–2014 for analysis. The NHANES program began in the early 1960s and is conducted as a series of surveys focusing on different health topics [16]. The sample for the NHANES survey was selected to represent the non-institutionalized United States population. Further information on background, design, and operation is available on the NHANES website (https://www.cdc.gov/nchs/nhanes/about_nhanes.htm).

### Ethics statement

All NHANES data was de-identified, and all participants provided written informed consent, consistent with approval by the National Center for Health Statistics Institutional Review Board. Institutional Review Board approval and informed consent signed by participants were waived in the NHANES data analysis.

### Study subjects

**Fig. 1:**
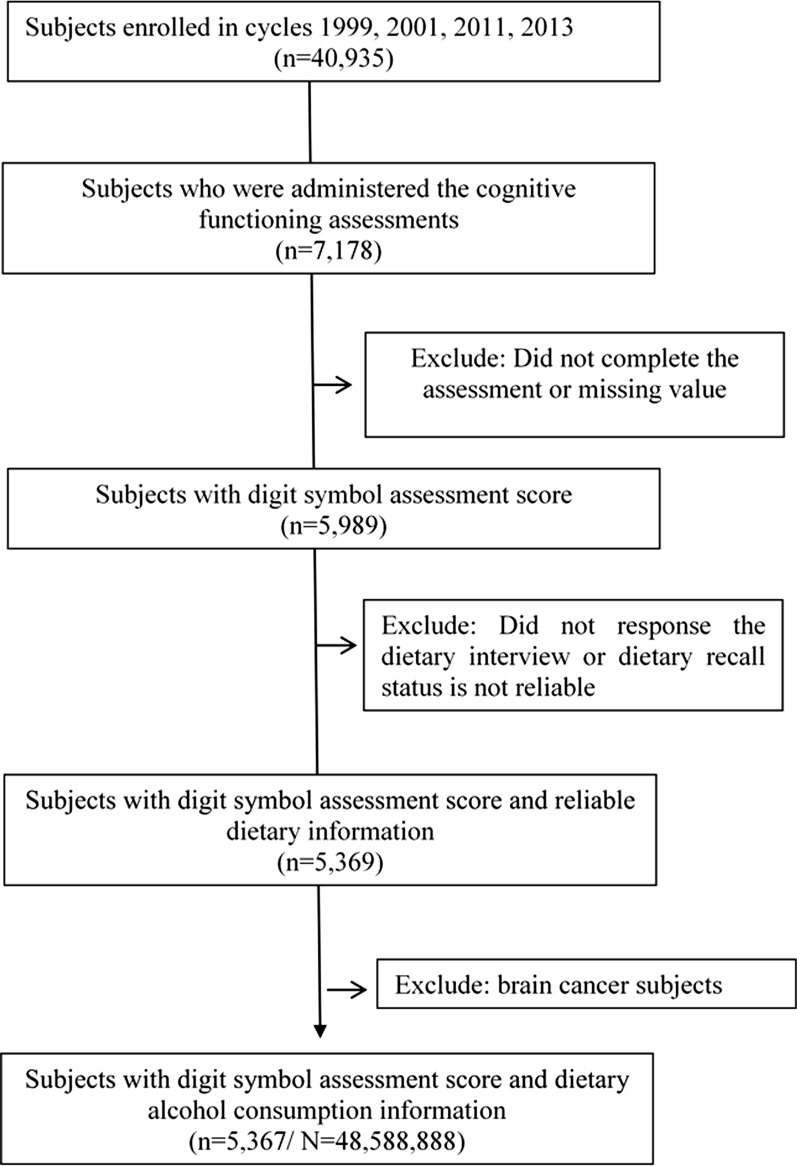
Flow chart of subject selection

Participants ≥ 60 years were administered the Digit Symbol Substitution Test (DSST) [15] to evaluate cognitive function and completed the dietary interview for enrollment in the present study. Subjects with incomplete DSST assessments or without alcohol consumption data were excluded. Subjects who had ever been diagnosed with brain cancer were also excluded. Figure 1 indicates the selection process.

### Study variables

#### Cognitive impairment

Cognitive impairment was defined as a DSST score below the lowest quartile. The DSST requires the study participant to code a series of symbols in 120 s with accuracy. This exercise needs response speed, sustained attention, visual-spatial skills, associative learning, and memory and is considered a sensitive measure of cognitive functioning [15].

#### Alcohol consumption

According to the Substance Abuse and Mental Health Services Administration (SAMHSA) criteria [17], alcohol consumption is classified into three groups: (1) Non-drinkers (0 g/day); (2) Drinkers (> 0 to < 70 g /day in males, > 0 to < 56 g /day in females); (3) Heavy drinkers (≥ 70 g /day in males, ≥ 56 g /day in females). Information on alcohol consumption was obtained from the dietary interview-individual foods files to estimate total energy intake, nutrients, and components of foods/beverages consumed 24 h before the interview. It records the alcohol intake of each subject in detail. One alcoholic drink equivalent [12 fluid ounces of regular beer (5% alcohol), 5 fluid ounces of wine (12% alcohol), or 1.5 fluid ounces of 80 proof distilled spirits (40% alcohol)] contains roughly 14 g of alcohol.

#### Demographic

We obtained information on age, sex, race/ethnicity, marital status, education level, and poverty income ratio (a ratio of family income to poverty threshold) from the NHANES database. Racial groups were non-Hispanic White, non-Hispanic Black, other Hispanic, Mexican American, and others (including multi-racial). Marital status was categorized as married/ living with a partner, widowed/ divorced/ separated, and never married. Educational status was grouped as under 12th grade, high school graduate, and college or above. Poverty income ratio were categorized as < 1 (below poverty level), 1–1.99, 2–3.99, ≥ 4 (richest).

#### Lifestyle

Smoking history was categorized as follows:

(i) Non-smokers: who had never smoked 100 cigarettes during their lifetime.
(ii) Smokers: who had smoked at least 100 cigarettes during their lifetime.

Data on physical activity was obtained using a physical activity questionnaire that included several questions on daily activities, leisure-time activities, and sedentary activities at home. Metabolic equivalent (MET) scores of activities were calculated for each subject [18]. Physical activity was classified into two groups: active (METs ≥ 500) and inactive (METs < 500). Body mass index (BMI, weight/height^2^) was calculated during participants’ physical examinations at the NHANES mobile examination center (MEC). According to the World Health Organization (WHO) criteria, BMI was classified into four groups: underweight (< 18.5 kg/m^2^), normal (18.5 ~ 24.9 kg/m^2^), overweight (25 ~ 29.9 kg/m^2^), and obese (≥ 30.0 kg/m^2^). Difficulties attending social events were self-reported on functional limitations and defined by the question “By yourself and without using any special equipment, how much difficulty do you have to participate in social activities [visiting friends, attending clubs, meetings or parties]?” Attending social events was divided into no difficulty and difficulty (including some difficulty, much difficulty, and unable to do).

#### Comorbidities

Comorbidities, including diabetes mellitus (DM), hypertension, and stroke, were self-reported by participants using NHANES interviewer-administered questionnaires and defined by the question “Have you ever been told by a doctor or other health professional that you had …?” Chronic kidney disease (CKD) was defined by urine albumin and creatinine ratio (ACR) ≥ 30 mg/g.

### Statistical analysis

Data on basic characteristics were expressed as unweighted counts (weighted %) for categorical variables, mean ± standard error for continuous variables. Likelihood ratio tests were used to determine the differences in categorical variables, and differences in continuous variables were examined using the Complex Samples General Linear Model (CSGLM). Univariate and multivariate logistic regression analyses were performed to explore associations between cognitive impairment and alcohol consumption. Variables with a significance level of < 0.05 by univariate analysis were selected and evaluated using a multivariate logistic regression model. Interaction analysis was performed between alcohol consumption and related factors. We analyzed subgroups stratified by age to explore the relationship between alcohol consumption and related factors associated with cognitive impairment in different age groups. To better delineate the dose–response relationship between alcohol consumption and cognitive impairment, the study subjects were stratified by number of alcoholic drinks (one drink = 14 g of alcohol) and categorized into 0, 1 to 2, 3 to 4, 5 to 6, 7 to 8, 9 to 10, and 10 + drinks. Odds ratios (OR) and 95% confidence intervals (CI) were depicted with different models. All analyses included sample dietary weight (WTDR4YR for 1999–2002, WTDRD1 for 2011–2014), stratum, and primary sampling units (PSU) per recommendations from the National Center for Health Statistics (NCHS) for a complex sampling design analysis to address oversampling, non-response, non-coverage and provide nationally representative estimates. All statistical assessments were two-sided and evaluated at the 0.05 level of significance. Statistical analyses were performed using a statistical software package, SPSS complex sample module version 22.0 (IBM Corp, Armonk, NY).

## Results

### Study population characteristics

A total of 5367 participants were eligible for the present study. Using the NHANES sample weight, the analytic sample size in the present study represented 48,588,888 non-institutionalized participants from the United States. We identified 1958 (36.4%) subjects with cognitive impairment. The basic characteristics of the study subjects are shown in Table 1. We observed a higher percentage of non-drinkers (84.2% versus 73.1%) and lower alcohol consumption (4.62 ± 0.518 versus 7.44 ± 0.413 g/day) in the cognitive impairment groups. Compared with the non-cognitive impairment group, participants with cognitive impairment were more likely to be older, male, non-Hispanic Black, widowed/ divorced/ separated, with lower education levels, poor income ratio, inactive lifestyle, and difficulties attending social events. Participants with cognitive impairment were more susceptible to comorbidities, such as DM, hypertension, stroke, and CKD (all p < 0.05).

**Table 1:** Characteristics of participants (Unweighted sample sizes and weighted %) according to cognitive impairment

Variables	Cognitive impairment (n = 1958/N = 11,422,175)	Non- Cognitive impairment (n = 3409/N = 37,166,712)	P-value
Alcohol consumption			
Alcohol (gram/day), mean ± SE	4.62 ± 0.518	7.44 ± 0.413	< 0.001
Non-drinkers	1659 (84.2)	2580 (73.1)	< 0.001
Drinkers	266 (14.2)	771 (25.1)	
Heavy drinkers	33 (01.6)	58 (01.9)	
Demographic			
Age, years			< 0.001
70+	1142 (66.0)	1498 (40.0)	
60–69	816 (34.0)	1911 (60.0)	
Gender			0.032
Male	1079 (48.3)	1556 (43.6)	
Race			< 0.001
Others (including multi-racial)	68 (03.2)	230 (04.4)	
Other Hispanic	217 (09.3)	163 (02.4)	
Mexican American	413 (07.0)	340 (02.1)	
Non-Hispanic Black	548 (17.4)	488 (05.0)	
Non-Hispanic White	712 (63.2)	2188 (86.0)	
Education			< 0.001
Under 12th grade	1142 (48.9)	594 (13.1)	
High school graduate	412 (25.5)	864 (25.2)	
College or above	398 (25.5)	1950 (61.8)	
Marital status			< 0.001
Married/Living with partner	1003 (52.7)	2143 (68.8)	
Widowed/Divorced/Separated	808 (43.1)	1061 (27.9)	
Never married	90 (04.2)	134 (03.4)	
Income ratio			< 0.001
< 1 (Below poverty level)	490 (23.9)	297 (06.3)	
1–1.99	718 (40.1)	746 (20.2)	
2–3.99	366 (22.9)	1001 (32.7)	
4–5 (richest)	186 (13.2)	1058 (40.7)	
Lifestyle			
Smoking			0.466
Current smoker	281 (12.5)	381 (11.0)	
Former smoker	755 (39.8)	1375 (40.0)	
Non-smoker	919 (47.7)	1649 (49.0)	
Physical activity			< 0.001
Active	491 (32.7)	1365 (51.8)	
Inactive	839 (67.3)	1282 (48.2)	
BMI			0.155
Underweight	36 (02.3)	38 (01.3)	
Normal	498 (28.0)	865 (25.9)	
Overweight	699 (35.8)	1266 (37.5)	
Obese	635 (33.9)	1183 (35.3)	
Attend social events			< 0.001
Difficulty	372 (21.3)	295 (07.3)	
No difficulty	1516 (78.7)	3075 (92.7)	
Comorbidities			
Diabetes mellitus	575 (28.0)	702 (18.0)	< 0.001
Hypertension	1220 (65.0)	1856 (51.7)	< 0.001
Stroke	207 (11.5)	149 (04.4)	< 0.001
CKD	428 (30.9)	397 (13.5)	< 0.001

SE: Standard Error; BMI: Body Mass Index; CKD: Chronic Kidney Disease

### Factors associated with cognitive impairment

Univariate logistic regression analyses revealed that alcohol consumption, age, race, education level, marital status, income ratio, physical activity, difficulties attending social events, presence of DM, hypertension, stroke, and CKD comorbidities were significantly associated with the risk of cognitive impairment (Table 2). After adjusting these significant factors, the results of multivariate logistic regression indicated that heavy drinker versus drinker had significantly negative association with the risk of cognitive impairment (aOR = 0.330, 95% CI 0.131–0.832). Moreover, the following factors showed a significant positive association with cognitive impairment: age (70+ y vs. 60–69 y, aOR = 6.501, 95% CI 3.076–13.74), male gender, race, education, income ratio, difficulties attending social events, hypertension, and CKD. The interaction term for alcohol consumption and age in the multivariate model was significant (heavy drinker*age, aOR = 8.052, 95% CI 1.167–55.54, non-drinker*age, aOR = 0.486, 95% CI 0.243–0.972). Due to the interaction effect of age on alcohol consumption, we performed a subgroup analysis stratified by age.

**Table 2:** Univariate and multivariate logistic regression analyses of risk variables for association with cognitive impairment

Variables	Univariate OR (95% CI)	Multivariate aOR (95% CI)
Alcohol consumption		
Non-drinkers (Ref = Drinkers)	2.028 (1.616–2.546)	1.531 (0.889–2.635)
Heavy drinkers	1.512 (0.800–2.859)	0.330 (0.131–0.832)
Drinkers (Ref = Non-drinkers)	0.493 (0.393–0.619)	0.653 (0.380–1.124)
Heavy drinker	0.746 (0.387–1.437)	0.216 (0.086–0.542)
Demographic		
Age, years (Ref = 60–69 y)		
70+	2.922 (2.444–3.493)	6.501 (3.076–13.74)
Gender (Ref = female)		
Male	1.208 (1.017–1.434)	1.826 (1.334–2.500)
Race (Ref = Non-Hispanic white)		
Others	0.975 (0.657–1.446)	1.042 (0.563–1.928)
Other Hispanic	5.292 (3.641–7.690)	4.352 (2.123–8.922)
Mexican American	4.485 (3.523–5.709)	3.741 (2.093–6.688)
Non-Hispanic black	4.708 (3.801–5.831)	5.965 (4.171–8.530)
Education (Ref = College or above)		
Under 12th grade	9.069 (7.493–10.97)	5.075 (3.506–7.346)
High school graduate	2.458 (1.978–3.056)	2.004 (1.501–2.675)
Marital status (Ref = Married/Living with partner)		
Widowed/Divorced/Separated	2.019 (1.698–2.400)	1.205 (0.855–1.699)
Never married	1.641 (1.105–2.438)	1.561 (0.666–3.662)
Income ratio (Ref = 4–5 (richest))		
< 1 (Below poverty level)	11.634 (8.362–16.18)	3.077 (1.804–5.251)
1–1.99	6.107 (4.516–8.260)	2.358 (1.549–3.591)
2–3.99	2.150 (1.571–2.944)	1.144 (0.720–1.816)
Lifestyle		
Smoking (Ref = Non-smoker)		
Current smoker	1.166 (0.908–1.497)	
Former smoker	1.021 (0.868–1.200)	
Physical activity (Ref = Active)		
Inactive	2.208 (1.824–2.671)	1.332 (0.966–1.837)
BMI^b^ (Ref = Normal)		
Underweight	1.659 (0.971–2.832)	
Overweight	0.887 (0.706–1.114)	
Obese	0.892 (0.718–1.108)	
Attend social events (Ref = No difficulty)		
Difficulty	3.405 (2.785–4.163)	2.876 (1.810–4.568)
Comorbidities (Ref = Without)		
Diabetes Mellitus	1.770 (1.479–2.119)	1.156 (0.734–1.819)
Hypertension	1.731 (1.473–2.034)	1.554 (1.171–2.063)
Stroke	2.820 (2.150–3.700)	1.470 (0.752–2.874)
CKD	2.864 (2.320–3.536)	2.200 (1.455–3.326)
Interaction term		
Heavy drinker*Age		8.052 (1.167–55.54)
Non-drinker*Age		0.486 (0.243–0.972)

Ref: reference; BMI: Body Mass Index; CKD: Chronic Kidney Disease

### Subgroup analysis

In Table 3, results of multivariate logistic regression in the young-old (60–69 years) subgroup indicated that heavy drinkers were significantly negatively associated with the risk of cognitive impairment (aOR: 0.280, 95% CI 0.095–0.826). Male gender, race, education, income ratio, BMI, and comorbidities (hypertension, stroke, and CKD) were significantly positively associated with the risk of cognitive impairment.

**Table 3:** Multivariate logistic regression analyses of risk variables for association with cognitive impairment-stratified by age

Variables	Cognitive impairment	
	60–69 y	70+ y
	aOR (95% CI)	aOR (95% CI)
Alcohol consumption (Ref = Drinkers)		
Non-drinkers	1.500 (0.734–3.065)	0.865 (0.505–1.480)
Heavy drinkers	0.280 (0.095–0.826)	2.929 (0.624–13.74)
Demographic		
Gender (Ref = Female)		
Male	2.696 (1.537–4.730)	1.733 (1.132–2.654)
Race (Ref = Non-Hispanic white)		
Others (including multi-racial)	1.019 (0.371–2.799)	0.888 (0.432–1.823)
Other Hispanic	4.604 (1.395–15.19)	4.941 (1.997–12.22)
Mexican American	4.678 (2.112–10.35)	2.447 (1.209–4.951)
Non-Hispanic black	8.041 (4.436–14.57)	5.180 (3.117–8.610)
Education (Ref = College or above)		
Under 12th grade	16.91 (8.669–33.00)	3.279 (2.046–5.254)
High school graduate	5.132 (2.587–10.18)	1.399 (0.973–2.010)
Marital status (Ref = Married/ Living with partner)		
Widowed/Divorced/Separated	0.920 (0.513–1.652)	1.379 (0.889–2.139)
Never married	2.644 (0.922–7.584)	1.063 (0.255–4.435)
Income ratio (Ref = 4–5 (richest))		
< 1 (Below poverty level)	2.782 (1.177–6.579)	2.329 (1.031–5.257)
1–1.99	2.960 (1.302–6.728)	1.940 (1.136–3.312)
2–3.99	0.846 (0.339–2.111)	1.242 (0.690–2.237)
Lifestyle		
Smoking (Ref = Non-smoker)		
Current smoker	0.890 (0.440–1.800)	0.724 (0.313–1.675)
Former smoker	1.049 (0.579–1.900)	0.814 (0.546–1.215)
Physical activity (Ref = Active)		
Inactive	1.102 (0.629–1.930)	1.674 (1.091–2.569)
BMI (Ref = Normal)		
Underweight	22.56 (4.054–125.5)	1.493 (0.450–4.951)
Overweight	0.542 (0.241–1.218)	0.548 (0.365–0.823)
Obese	0.499 (0.269–0.925)	0.496 (0.264–0.932)
Attend social events (Ref = No difficulty)		
Difficulty	2.256 (0.600–8.482)	2.817 (1.547–5.132)
Comorbidities (Ref = Without)		
Diabetes mellitus	0.930 (0.496–1.745)	1.502 (0.937–2.408)
Hypertension	1.923 (1.155–3.202)	1.707 (1.160–2.513)
Stroke	4.823 (1.527–15.23)	1.285 (0.707–2.334)
CKD	3.121 (1.337–7.287)	1.835 (1.052–3.202)

Ref: reference; BMI: Body Mass Index; CKD: Chronic Kidney Disease

In the middle-old (≧70 years) subgroup, heavy drinking was associated with a higher risk of cognitive impairment (aOR: 2.929, 95% CI 0.624–13.74). Male gender, race, education, income ratio, inactive lifestyle, BMI, difficulty attending social events, comorbidities (hypertension and CKD) were positively associated with cognitive impairment.

### J-shaped association between alcohol consumption and cognitive impairment

**Fig. 2:**
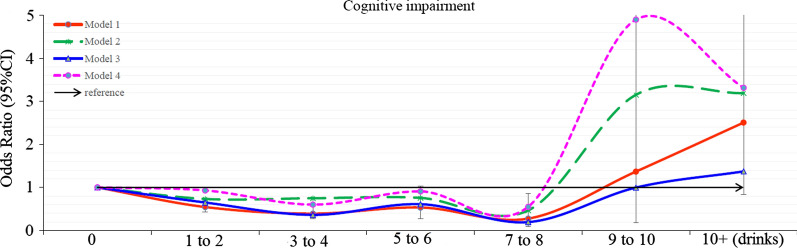
Odds ratio for cognitive impairment by different alcohol consumption categories. Model 1: crude odds ratio; Model 2: adjusted for demographic variables; Model 3: adjusted for comorbidity variables; Model 4: adjusted for all significant variables in the Table 2. Error bars depict 95% CI

Additional file 1: Table S1 describes the characteristics of study subjects stratified by 2 alcoholic drinks. DSST scores increased with an increase in the alcohol dose, up to 3 to 4 drinks, and then gradually decreased. Participants consuming more alcohol were young-elderly, male, non-Hispanic Black, inactive, overweight, or obese. As compared to non-drinkers (0 drink), 1 to 2, 3 to 4, 5 to 6, 7 to 8 drinks had decreased risk of cognitive impairment (OR = 0.545, 0.391, 0.534, and 0.276, respectively) (Additional file 1: Table S2). There was a slight J-shaped association between alcohol intake and cognitive impairment (Fig. 2). This association remained consistent even after adjusting for demographic variables (Model 2), comorbidity variables (Model 3), and all significant variables in Table 2 (Model 4).

**Fig. 3:**
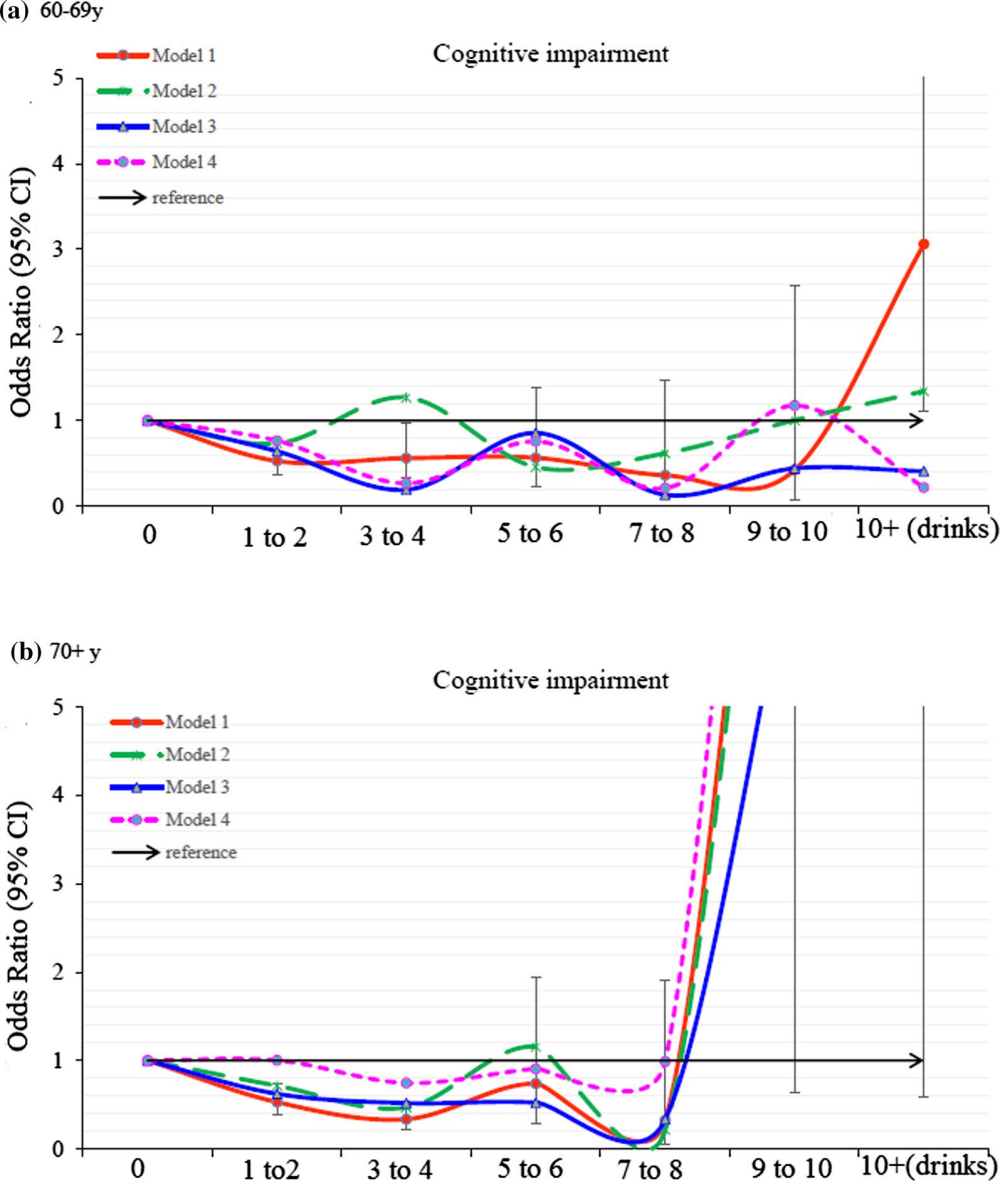
Odds ratio for cognitive impairment by different alcohol consumption categories- stratified by age. Model 1: crude odds ratio; Model 2: adjusted for demographic variables; Model 3: adjusted for comorbidity variables; Model 4: adjusted for all significant variables in the Table 2. Error bars depict 95% CI

To clarify the role of age in the relationship between alcohol drinking and cognitive function, we further examined the dose–response relationship in different age groups. In the young-old (60–69 years) subgroup, compared to 0 drink, 1 to 2, 3 to 4, 5 to 6, 7 to 8, 9 to 10 drinks had decreased risk of cognitive impairment (OR = 0.528, 0.561, 0.565, 0.361, and 0.423, respectively). However, after adjusting for other confounders, the slight J-shaped association was no longer consistent. In contrast, the J-shaped association was profound and consistent for those over 70 years. Compared to 0 drink, 1 to 2, 3 to 4, 5 to 6, 7 to 8 drinks had decreased risk of cognitive impairment (OR = 0.529, 0.336, 0.739, and 0.324, respectively). This association was consistent after adjusting for other confounders (Additional file 1: Table S3, Fig. 3).

## Discussion

This study demonstrated that heavy alcohol drinking in young-old people (60–69 years) was associated with a lower risk of cognitive impairment, while middle-old people (≧70 years) showed a higher risk of cognitive impairment than non-drinkers. This study also revealed that older adults (≧70 years), men, people from minority races, persons with lower education or income levels, people with difficulty attending social events, and those with hypertension or CKD were significantly associated with a higher risk of cognitive impairment.

Cognition refers to the mental functions involved in learning, memory, judgment, expression, and work [1, 15]. Some researchers have reported that older adults may be more susceptible to cognitive dysfunction due to brain aging and long-term cumulative injury [15, 19]. People with lower education levels may have less brain reserve and a higher risk of cognitive decline [1, 15, 20]. Ethnic minorities and people with low income or social difficulties and an inability to afford good food may have increased susceptibility to cognitive dysfunction [1, 15, 21]. People with hypertension or chronic kidney disease may have long-term inflammation and oxidative stress with a higher risk of cognitive impairment [1]. Men may experience higher stress in daily activities without the protective effects of estrogen (seen in women), leading to a higher risk of cognitive decline [1, 15]. Our study also revealed that the abovementioned factors were associated with a higher risk of cognitive impairment.

Studies have shown that a low or moderate amount of alcohol consumption protects against cognitive impairment [4, 7, 8]. However, some studies show that low or moderate alcohol drinking has a neutral effect or a higher risk of cognitive impairment [9–11, 22]. The conflicting results could be because these studies used different tools to evaluate cognitive dysfunction; the definitions of the quantity and pattern of alcohol drinking, smoking status, educational and occupational attainment, comorbidities, and psychotropic drugs use of the drinkers and non-drinkers were different [4, 11]. However, the recent studies have indicated that a low or moderate amount of alcohol consumption is associated with a lower risk of cognitive impairment [23]. These findings are consistent with our study result that low to moderate alcohol consumption was associated with a lower risk of cognitive impairment. The reasons are as follows: (1) low to moderate alcohol drinking can increase high-density lipoprotein (HDL) levels, decrease platelet aggregation, increase fibrinolysis, and inhibit thrombotic activity [24]; (2) alcohol drinking can reduce systemic inflammation and facilitate antioxidant effect [25]; (3) Moderate alcohol drinking was reported to increase the levels of brain-derived neurotrophic factor (a key regulator of neuronal plasticity and development) in the dorsal striatum [26]. However, alcohol consumption can lead to liver disease, accidents, stroke, and cancers [27].

Studies show that heavy alcohol drinking may have direct or indirect detrimental effects on the brain [4, 11, 28]. First, acetaldehyde and reactive oxygen species (ROS) produced by alcohol metabolism can have toxicological concerns to the liver, gut and brain [5, 29, 30]. Alcohol metabolism can generate mitochondrial damage and hypoxia, make cells to undergo necrosis, apoptosis, and induce inflammation. ROS may lead to less antioxidants, breakdown of electron transport chain and reduced ATP production, mitochondrial membrane collapse and lysosomal membrane leakage, and ultimately cause cell injury or death [30]. ROS also can stimulate the activation of nuclear factor-κB (NF-κB) and increase inflammation [30]. Second, alcohol can modify the fluidity of cell membranes, interact with calcium and chlorine channels, and impair cell function. It acts on several neural networks served by different neurotransmitters [28]. Third, alcohol can also block *N*-methyl-d-aspartate (NMDA) receptors. Chronic inhibition of NMDA receptors can increase glutamate release with excitotoxic effects on neurons [28, 31]. Fourth, malnutrition, thiamine deficiency, and other vitamin deficiencies can cause indirect neuronal damage [28]. Fifth, heavy drinking can lead to hyperlipidemia, high blood pressure, increased risk of stroke, and brain injury [5]. Finally, chronic alcohol consumption can impair the microbiota balance and barrier function of the gut, increase lipopolysaccride (LPS) translocation, decrease liver’s ability to detoxify bacterial products and engender an imbalance in cytokine milieu, abate the brain’s ability to regulate periphery inflammation, and give rise to persistent systemic inflammation and organ damage [30]. However, there are still reports showing neutral or protective effects of heavy alcohol drinking against cognitive impairment [4, 11]. As we divided the older adults into young-old (60–69 years) and middle-old (≧70 years) groups, heavy alcohol drinking seemed to provide protective effects against cognitive impairment in young-old persons, with a higher risk of cognitive impairment in middle-old persons. But we must be careful that this is a cross-sectional study, we can only see association but not causality. Young-old people with heavy alcohol drinking may have be more financially capable. They can have a healthy diet with appropriate leisure activities, which can lead them to less cognition decline. It is also possible that the DSST may not be sensitive enough to detect cognitive impairment caused by heavy drinking in this study. Older adults showing an accumulation of various brain insults with time may be more vulnerable to the effects of heavy alcohol drinking [15, 18, 32, 33]. However, many older adults may have the habit of alcohol drinking [34] and it will be challenging to influence drinking behavior when there is long interval between risk-taking behaviors and the onset of complications. We should stress the importance of restricting alcohol use in middle-old people to decrease the risk of cognitive impairment.

Studies have demonstrated that the use of probiotics can improve gut permeability and attenuate tissue injury in patients or animals with alcoholic liver diseases. We may suggest probiotics for older people with heavy drink to potentially mitigate inflammation [30]. Oxidative stress occurs when the endogenous antioxidant defenses are unable to eliminate excessive ROS. Older adults have insufficient antioxidant defense and may not be able to eliminate the excess production of ROS by heavy drinking with the susceptibility to cognitive decline [35]. Eftekhari and Ahmadian, et al. have performed exquisite researches to demonstrate that antioxidants (*N*-acetylcysteine, quercetine, and taurine), ROS scavengers (a-tocopherol succinate and/or butylated hydroxyltoluene), ATP generators (fructose and/r l-glutamine), mitochondrial permeability transition (MPT) pore sealing agents (carnitine and/or trifluoperazine), endocytosis inhibitors (chloroquine and/or methylamine), and CYP450 inhibitors (4-methylpyrazole and/or cimetidine) can prevent medication-induced oxidative stress cytotoxicity [36, 37]. We may advise the elderly to consume more unprocessed vegetables, fruits, fish and meats to increase the natural antioxidants. But because the older people have bad teeth. Their absorption of nutrients is not good. They may need to supplement antioxidant medications. Due to poor water solubility, slow permeability, gastrointestinal degradation, first-pass effect, and instability during storage, most antioxidants have not been used successfully. This problem may be solved by adopting the nanomedicine, that is to encapsulate or process the antioxidants as nanoparticles. These options can increase the solubility, permeability and preservation of nano-antioxidants, also enhance their surface area, uptake and transport to the target sites; which will work to improve intracellular penetration and distribution of the nano-antioxidants. Nanocapsulated quercetin (a flavonoid) has been demonstrated to reduce the oxidative stress of brain damage caused by arsenic exposure [38]. We may suggest older people to take nano-antioxidants to plummet the oxidative stress and combat the cognitive dysfunction caused by heavy alcohol drinking.

Some limitations in this study need to be stated. First, as NHANES data analysis was cross-sectional in design, we could not determine the duration, pattern, and frequency of alcohol drinking. The assessment of alcohol consumption was based on self-report instead of objective measures, and therefore, may be subject to bias; however, current evidence reveals that this may be a reliable and valid approach to measure alcohol consumption [39]. Second, the NHANES examined persons dwelling in the community, not institutions. The community-dwelling older adults may be healthier than nursing home residents; therefore, our results may underestimate the risk of cognitive impairment. Third, we excluded subjects who could not complete DSST assessments, or those individuals who could not enroll in surveys. Thus, those who participated in the NHANES were relatively healthy or resistant to the toxic effects of alcohol. Fourth, we used the DSST to assess cognitive impairment, which is sensitive to cognitive decline [15]. However, this test alone may not provide insights into different domains of cognitive processing. Fifth, this dataset lacks information on apolipoprotein Eɛ4 (APOE E4) [24], alcohol dehydrogenase 1B (ADH1B), and acetaldehyde dehydrogenase 2 (ALDH2) genes, and therefore, we did not include these 3 genes as variables in the analysis. Sixth, the results of this study apply to the American population and may not apply to other countries. Finally, this is a cross-sectional study with some inevitable bias. Prospective randomized control trials are needed to verify our results.

## Conclusion

Studies on the cognitive repercussions of alcohol drinking by using real world national represented database are scarce in the literature. A better understanding of the differences in the impact of alcohol use on cognitive impairment in older adults may provide valuable information on their care. Our study showed that light to heavy drinking was associated with lower risk of cognitive impairment in young old, heavy alcohol drinking was associated with a higher risk of cognitive impairment in middle-old people. We recommend that older adults with drinking habits restrict alcohol use to attenuate the risk of cognitive impairment or dementia in the future.

## Supplementary Information

**Additional file 1: Table S1. **Characteristics of Participants (Unweighted sample sizes and weighted %) according to alcohol consumption (gram/day). **Table S2.** Odds ratio for cognitive impairment by different alcohol consumption categories. **Table S3.** Odds ratio for cognitive impairment by different alcohol consumption categories-stratified by age

## Data Availability

Data from the National Health and Nutrition Examination Survey (NHANES) 1999–2002, 2011–2014 were used for this analysis. Further information on background, design, and operation is available on the NHANES website (https://www.cdc.gov/nchs/nhanes/about_nhanes.htm).

## Abbreviations

DSST: Digit symbol substitution test
MET: Metabolic equivalent
CKD: Chronic kidney disease
DM: Diabetes mellitus
NMDA: *N*-methyl-d-aspartate
