# Supplementary material for: The impact of heavy alcohol consumption on cognitive impairment in young old and middle old persons
Source: J Transl Med. 2022 Apr 5;20:155. doi: 10.1186/s12967-022-03353-3 (PMC8981936; doi:10.1186/s12967-022-03353-3)
Supplement: Supplementary file 1 — Additional file 1: Table S1. Characteristics of Participants (Unweighted sample sizes and weighted %) according to alcohol consumption (gram/day). Table S2. Odds ratio for cognitive impairment by different alcohol consumption categories. Table S3. Odds ratio for cognitive impairment by different alcohol consumption categories-stratified by age [file 12967_2022_3353_MOESM1_ESM.docx]

Supplementary material

**Table S1** Characteristics of Participants (Unweighted sample sizes and weighted %) according to alcohol consumption (gram/day)

| Alcohol consumption, drink | Total | 0 | 1 to 2 | 3 to 4 | 5 to 6 | 7 to 8 | 9 to 10 | >10 | P-value |
| --- | --- | --- | --- | --- | --- | --- | --- | --- | --- |
| No of subjects | 5367 | 4239 | 726 | 264 | 90 | 21 | 8 | 19 |  |
| Alcohol (gram/day), mean±SE | 6.78±0.34 | 0±0.00 | 13.06±0.32 | 38.85±0.52 | 67.78±1.14 | 95.31±2.19 | 119.6±2.17 | 173.9±8.89 | <0.001 |
| Cognitive function |  |  |  |  |  |  |  |  |  |
| Impairment | 1958 (23.5) | 1659 (26.1) | 193 (16.2) | 61 (12.1) | 25 (15.9) | 6 (8.9) | 3 (32.6) | 11 (47.0) | <0.001 |
| DSST^a^ score | 49.93±0.47 | 48.31±0.55 | 54.39±0.78 | 57.13±1.21 | 54.33±1.91 | 55.32±4.54 | 44.97±5.44 | 43.81±2.97 | <0.001 |
| Demography |  |  |  |  |  |  |  |  |  |
| Age, years |  |  |  |  |  |  |  |  | <0.001 |
| 70+ | 2640 (46.1) | 2113 (47.5) | 381 (47.6) | 104 (33.8) | 31 (29.2) | 7 (24.4) | 2 (31.7) | 2 (19.5) |  |
| 60-69 | 2727 (53.9) | 2126 (52.5) | 345 (52.4) | 160 (66.2) | 59 (70.8) | 14 (75.6) | 6 (68.3) | 17 (80.5) |  |
| Gender |  |  |  |  |  |  |  |  | <0.001 |
| Male | 2635 (44.7) | 1942 (41.4) | 397 (47.0) | 178 (62.5) | 76 (80.8) | 17 (79.7) | 8 (100) | 17 (88.4) |  |
| Race |  |  |  |  |  |  |  |  | 0.008 |
| Others (including multi-racial) | 298 (4.1) | 248 (4.4) | 43 (4.4) | 6 (1.6) | 1 (0.3) | 0 (0.0) | 0 (0.0) | 0 (0.0) |  |
| Other Hispanic | 380 (4.0) | 313 (4.4) | 46 (3.1) | 12 (2.0) | 5 (1.6) | 3 (4.6) | 0 (0.0) | 1 (3.6) |  |
| Mexican American | 753 (3.3) | 636 (3.6) | 79 (2.2) | 26 (2.1) | 9 (2.7) | 0 (0.0) | 1 (2.2) | 2 (4.3) |  |
| Black | 1036 (7.9) | 870 (8.9) | 97 (4.8) | 40 (4.0) | 14 (5.3) | 3 (2.5) | 4 (16.1) | 8 (23.9) |  |
| White | 2900 (80.6) | 2172 (78.6) | 461 (85.4) | 180 (90.3) | 61 (90.1) | 15 (92.9) | 3 (81.8) | 8 (68.2) |  |
| Education |  |  |  |  |  |  |  |  | <0.001 |
| Under 12th grade | 1736 (21.5) | 1491 (24.8) | 165 (12.1) | 50 (9.5) | 20 (13.3) | 2 (1.3) | 3 (8.9) | 5 (17.7) |  |
| High school graduate | 1276 (25.2) | 1033 (26.1) | 160 (25.5) | 48 (14.4) | 21 (26.1) | 6 (28.6) | 1 (27.3) | 7 (25.0) |  |
| College or above | 2348 (53.3) | 1710 (49.2) | 399 (62.4) | 166 (76.1) | 49 (60.6) | 13 (70.1) | 4 (63.8) | 7 (57.3) |  |
| Marital status |  |  |  |  |  |  |  |  | 0.008 |
| Married/ Living with partner | 3146 (65.0) | 2419 (62.8) | 472 (71.0) | 176 (74.5) | 54 (69.0) | 13 (82.6) | 5 (67.4) | 7 (52.1) |  |
| Widowed/ Divorced/ Separated | 1869 (31.4) | 1547 (33.4) | 204 (26.1) | 71 (23.7) | 28 (25.1) | 5 (14.9) | 3 (32.6) | 11 (45.2) |  |
| Never married | 224 (3.6) | 177 (3.8) | 27 (2.9) | 10 (1.8) | 7 (5.9) | 2 (2.5) | 0 (0.0) | 1 (2.7) |  |
| Income ratio |  |  |  |  |  |  |  |  | <0.001 |
| <1 | 787 (10.4) | 679 (11.8) | 70 (7.7) | 18 (2.4) | 10 (04.5) | 3 (2.9) | 0 (0.0) | 7 (29.3) |  |
| 1-1.99 | 1464 (24.9) | 1235 (27.2) | 160 (19.6) | 40 (12.6) | 22 (22.5) | 1 (3.3) | 2 (9.9) | 4 (13.4) |  |
| 2-3.99 | 1367 (30.4) | 1072 (30.9) | 192 (30.5) | 70 (26.0) | 21 (24.9) | 7 (33.4) | 2 (44.8) | 3 (26.6) |  |
| 4-5 (richest) | 1244 (34.3) | 857 (30.0) | 228 (42.3) | 113 (59.0) | 29 (48.1) | 10 (60.4) | 3 (45.3) | 4 (30.7) |  |
| **Lifestyle** |  |  |  |  |  |  |  |  |  |
| Smoking |  |  |  |  |  |  |  |  | <0.001 |
| Current smoker | 662 (11.3) | 482 (10.9) | 82 (8.9) | 56 (15.6) | 27 (29.4) | 5 (17.9) | 2 (5.3) | 8 (32.7) |  |
| Former smoker | 2130 (40.0) | 1594 (37.0) | 335 (46.3) | 139 (58.0) | 36 (36.0) | 12 (67.3) | 6 (94.7) | 8 (49.2) |  |
| Non-smoker | 2568 (48.7) | 2159 (52.1) | 307 (44.8) | 68 (26.4) | 27 (34.6) | 4 (14.8) | 0 (00.0) | 3 (18.2) |  |
| Physical activity |  |  |  |  |  |  |  |  | <0.001 |
| Active | 1856 (47.8) | 1369 (44.9) | 318 (58.5) | 116 (51.9) | 36 (53.1) | 10 (49.5) | 0 (0.0) | 7 (47.9) |  |
| Inactive | 2121 (52.2) | 1715 (55.1) | 243 (41.5) | 105 (48.1) | 34 (46.9) | 8 (50.5) | 6 (100.) | 10 (52.1) |  |
| BMI^b^ |  |  |  |  |  |  |  |  | <0.001 |
| Underweight | 74 (1.5) | 56 (1.5) | 12 (1.9) | 1 (0.5) | 3 (1.4) | 1 (1.2) | 0 (0.0) | 1 (1.3) |  |
| Overweight | 1965 (37.1) | 1523 (36.1) | 275 (38.2) | 109 (43.3) | 40 (44.2) | 10 (47.5) | 2 (6.6) | 6 (46.1) |  |
| Obese | 1818 (35.0) | 1526 (38.1) | 186 (24.6) | 67 (26.3) | 24 (26.2) | 5 (25.7) | 4 (88.1) | 6 (23.3) |  |
| Normal | 1363 (26.4) | 1004 (24.3) | 242 (35.4) | 81 (29.9) | 23 (28.2) | 5 (25.6) | 2 (5.3) | 6 (29.3) |  |
| Attend social events |  |  |  |  |  |  |  |  | <0.001 |
| Difficulty | 667 (10.6) | 588 (12.1) | 49 (6.4) | 18 (3.9) | 7 (6.9) | 2 (5.1) | 0 (0.0) | 3 (7.9) |  |
| No difficulty | 4591 (89.4) | 3558 (87.9) | 669 (93.6) | 243 (96.1) | 80 (93.1) | 18 (94.9) | 8 (100.) | 15 (92.1) |  |
| Comorbidity |  |  |  |  |  |  |  |  |  |
| Diabetes Mellitus | 1277 (20.3) | 1126 (23.2) | 106 (13.6) | 27 (7.7) | 11 (8.3) | 3 (4.9) | 2 (10.8) | 2 (5.9) | <0.001 |
| Hypertension | 3076 (54.9) | 2483 (56.4) | 388 (50.3) | 132 (49.8) | 44 (43.3) | 15 (70.4) | 6 (93.3) | 8 (43.9) | 0.009 |
| Stroke | 356 (6.1) | 301 (6.8) | 44 (5.3) | 6 (1.8) | 1 (0.4) | 1 (1.3) | 2 (29.6) | 1 (2.9) | 0.001 |
| CKD^c^ | 825 (17.5) | 677 (18.7) | 100 (14.3) | 28 (12.7) | 16 (15.2) | 1 (0.6) | 1 (28.6) | 2 (12.6) | 0.066 |

a. DSST: Digit Symbol Substitution Test

b. BMI: Body Mass Index

c. CKD: Chronic Kidney Disease.

**Table S2** Odds ratio for cognitive impairment by different alcohol consumption categories

| Alcohol consumption, drinks | Model 1 | Model 2 | Model 3 | Model 4 |
| --- | --- | --- | --- | --- |
|  | **OR (95%CI)** | **aOR (95%CI)** | **aOR (95%CI)** | **aOR (95%CI)** |
| 0 | Reference | Reference | Reference | Reference |
| 1 to 2 | 0.545 (0.426-0.696) | 0.733 (0.549-0.978) | 0.650 (0.461-0.914) | 0.931 (0.618-1.402) |
| 3 to 4 | 0.391 (0.273-0.559) | 0.749 (0.440-1.277) | 0.362 (0.232-0.566) | 0.601 (0.335-1.078) |
| 5 to 6 | 0.534 (0.278-1.027) | 0.757 (0.390-1.471) | 0.611 (0.296-1.258) | 0.908 (0.384-2.149) |
| 7 to 8 | 0.276 (0.089-0.858) | 0.467 (0.178-1.225) | 0.195 (0.036-1.053) | 0.552 (0.099-3.083) |
| 9 to 10 | 1.368 (0.182-10.27) | 3.151 (0.493-20.14) | 0.996 (0.223-4.451) | 4.898 (1.247-19.23) |
| >10 | 2.506 (0.837-7.499) | 3.189 (0.644-15.77) | 1.373 (0.238-7.915) | 3.315 (0.322-34.13) |

Model 1: crude OR (95%CI); Model 2: adjusted for demographic variables; Model 3: adjusted for comorbidity variables; Model 4: adjusted for all significant variables in the Table 2.

**Table S3** Odds ratio for cognitive impairment by different alcohol consumption categories-stratified by age

| **Alcohol consumption,**  **drinks** | **60-69y** | | | | **70+ y** | | | |
| --- | --- | --- | --- | --- | --- | --- | --- | --- |
|  | **Model 1** | **Model 2** | **Model 3** | **Model 4** | **Model 1** | **Model 2** | **Model 3** | **Model 4** |
|  | **OR (95%CI)** | **aOR (95%CI)** | **aOR (95%CI)** | **aOR (95%CI)** | **OR (95%CI)** | **aOR (95%CI)** | **aOR (95%CI)** | **aOR (95%CI)** |
| 0 | Reference | Reference | Reference | Reference | Reference | Reference | Reference | Reference |
| 1 to 2 | 0.528 (0.373-0.747) | 0.733 (0.453-1.184) | 0.642 (0.418-0.987) | 0.765 (0.421-1.389) | 0.529 (0.383-0.731) | 0.715 (0.475-1.076) | 0.623 (0.412-0.941) | 1.002 (0.598-1.679) |
| 3 to 4 | 0.561 (0.325-0.970) | 1.270 (0.531-3.041) | 0.193 (0.095-0.391) | 0.267 (0.106-0.675) | 0.336 (0.221-0.511) | 0.469 (0.275-0.799) | 0.518 (0.308-0.872) | 0.746 (0.381-1.464) |
| 5 to 6 | 0.565 (0.232-1.378) | 0.457 (0.173-1.211) | 0.855 (0.305-2.397) | 0.757 (0.216-2.649) | 0.739 (0.282-1.937) | 1.157 (0.427-3.138) | 0.523 (0.164-1.663) | 0.907 (0.245-3.358) |
| 7 to 8 | 0.361 (0.088-1.480) | 0.618 (0.191-2.001) | 0.134 (0.013-1.366) | 0.212 (0.026-1.729) | 0.324 (0.055-1.909) | 0.217 (0.029-1.621) | 0.334 (0.042-2.653) | 0.990 (0.096-10.19) |
| 9 to 10 | 0.423 (0.070-2.575) | 1.004 (0.155-6.490) | 0.442 (0.070-2.781) | 1.175 (0.133-10.38) | 10.72 (0.629-182.7) | 11.21 (0.428-193.9) | 6.882 (0.437-109.6)^a^ | 15.02 (0.788-290.3)^a^ |
| >10 drinks | 3.060 (1.109-8.446) | 1.344 (0.381-4.743) | 0.408 (0.103-1.613) | 0.218 (0.065-0.737) | 9.969 (0.584-170.3) | 18.99 (0.883-408.7) | 13.43 (0.833-216.7) | 29.06 (1.480-570.6) |

a. Interpolated value

Model 1: crude OR (95%CI).

Model 2: adjusted for demographic variables.

Model 3: adjusted for comorbidity variables.

Model 4: adjusted for all significant variables in Table 2.
